# Supplementary material for: Network-based visualisation of frequent sequences
Source: PLoS One. 2024 May 9;19(5):e0301262. doi: 10.1371/journal.pone.0301262 (PMC11081390; doi:10.1371/journal.pone.0301262)
Supplement: Attachment — Submitted filename: NbVFS PLOS ONE review response letter.pdf [file pone.0301262.s001.pdf]

Dear Reviewers,

Thank you for your time and efforts to review our article. Based on your valuable comments and suggestions, amendments are made to the manuscript. These amendments surely increase the overall quality of our work. Please find the detailed answers to your findings below.

Reviewer #1:

1. Please underscore the scientific value added to your paper in your abstract and introduction.
Thank you for this suggestion. Indeed, the aim and added value of our work was not underlined properly. The abstract and introduction is extended now with this aspect.

2. Some more recent schemes are also available; how do authors justify that the methods employed are appropriate?
Thank you for this question. The introduction was extended and redrafted to show the existing techniques of sequence pattern visualisations, and to show the novelty and usefulness of our method by comparing it to existing methods based on three key attributes.

3. The most recent papers need to be addressed in the related work sections.
Thank you for this note, some recent works were added to the introduction.

4. What is the time complexity of proposed algorithms?
The input of the network are events that are connected. The number of events is |I|. The method assumes that sequence patterns are already gained. Complexity of sequence pattern mining is $2^{|I|}$. The network visualisation part can be split in two: the weighted network and the MDS representation. In the case of the weighted network, the maximal number of edges between |I| nodes is |I|*(|I|-1), that is the complexity. The complexity of the MDS-based representation depends mainly on the complexity of the MDS algorithm (which is equivalent to the complexity of the distance matrix), that is $|I|^3$. In the case of large amounts of events (where the method starts to be really helpful), the exponential characteristic of the sequence pattern mining becomes dominant, so the visualisation part does not cause significant complexity increase compared to the sequence pattern mining itself. The discussion of the complexity issue is presented now in the manuscript.

5. The motivation of the proposed method should be stated in the introduction.
Thank you, the motivation is emphasised more now.

6. The method proposed by the authors should be compared with the recent existing methods and explained to highlight the advantages of the new method.
Thank you for the suggestion, the difference between our method and the recent ones is discussed in the introduction now.

8. How much performance improved in terms of percentage? Please use the abstract to explain this precisely.
The method aims to increase the processing time of sequential pattern mining results and to support the selection of relevant related events in the case of subprocess exploration tasks. As it is hard to define a standard value for human cognitive abilities, this question cannot be answered properly. However, if the performance is measured by the information content, we can state that our method allows the visualisation of multiple information, it can significantly lower the time consumption of the post-process activity. We tried to emphasise more the application areas and benefits of the proposed method in the abstract and in the introduction.

9. The language needs to be polished, some grammatical mistakes need to correct.
10. Some formatting errors exist. Correct the entire manuscript with proper formatting.
Thank you for these recommendations, the entire manuscript was checked according to it.

11. The results are not enough to show the efficiency of the proposed work, so, please extend the results parts to include further experiments.
Thank you for this suggestion, we made efforts to emphasise more the benefits and application areas/conditions of the suggested method. We truly believe that the provided experimental results underline the scientific and real life application value of our work. The source code will be shared on GitHub to make the method available for the wide scientific community.

12. Use high quality images for better visibility.
Thank you for this note, Fig2 and Fig3 are reproduced in high quality now.

Reviewer #2: In this manuscript, authors aimed to extend excellent visualization-based by applying a network-based approach to frequent sequence pattern mining, seeking to fulfill the mentioned requirements as well as possible. Authors suggested to address the following comments and suggestions when preparing the revised version:

= Abstract: section needs to be re-drafted to be self-contained means it has to clearly show the hypothesis, methodology, techniques and tools used, and the results obtained.
Thank you for this note, the abstract is extended now to touch these topics.

= Keywords: Authors suggested to add some keywords by selecting relevant terms. Keywords play important role in the appearance of the manuscript in scholars search which will give it more hits and more citations.
We fully agree with this approach, unfortunately the PLOS ONE Latex template does not contain a keywords section.

= What assumptions authors made during the simulation phase of this research work? If there is any.
Thank you for the question. After analysing metrics of visualisation methods, we have chosen three attributes to grade our method: Information content, Readability and Flexibility. This aspect is emphasised more now in the Introduction.

= Authors suggested to go through the following references and they MAY make use of them in updating the introduction and the related work sections:
- Ali Akbar Movassagh, Jafar A. Alzubi, Mehdi Gheisari, Mohamadtaghi Rahimi, Senthil kumar Mohan, Aaqif Afzaal Abbasi, Narjes Nabipour, "Artificial neural networks training algorithm integrating invasive weed optimization with diferential evolutionary model" Journal of Ambient Intelligence Humanized Computing, https://doi.org/10.1007/s12652-020-02623-6
- Omar A. Alzubi, Jafar A. Alzubi, Mohammed Alweshah, Issa Qiqieh, Sara Al-Shami, Manikandan Ramachandran, "An Optimal Pruning Algorithm of Classifier Ensembles: Dynamic Programming Approach" Neural Computing & Applications, 2020.
- Jafar A. Alzubi "Diversity Based Improved Bagging Algorithm" In Proceedings of the International Conference on Engineering & MIS 2015 (ICEMIS '15). Istanbul - Turkey.
- Jafar A. Alzubi, "Diversity-Based Boosting Algorithm". International Journal of Advanced Computer Science and Applications, Vol. 7, No. 5, 2016.
- O. A. Alzubi, J. A. Alzubi, S.Tedmori, H. Rashaideh, and O. Almomani "Consensus-Based Combining Method for Classifier Ensembles" The International Arab Journal of Information Technology, Vol. 15, No. 1, 2018.
Thank you for the suggestions, one article was found useful and was added to the Introduction section.

= Conclusion: The conclusion should be abstracted so authors need to consider re-drafting it.
Thank you for the suggestion, the Conclusion is re-drafted now.

= Authors need to confirm that all acronyms are defined before being used for first time.
= Authors need to confirm that all mathematical notations are defined when being used for first time.
= Authors suggested to proofread the manuscript after addressing all comments to avoid any typo, grammatical, and lingual mistakes and errors.= Authors advised to make sure that the format of all references is matching and complying with journal requirements and format.

Thank you, the manuscript was double checked and amended regarding your suggestions.
