# Supplementary material for: Network-based visualisation of frequent sequences
Source: PLoS One. 2024 May 9;19(5):e0301262. doi: 10.1371/journal.pone.0301262 (PMC11081390; doi:10.1371/journal.pone.0301262)
Supplement: Attachment — Submitted filename: NbVFS PLOS ONE review response letter round 2.pdf [file pone.0301262.s002.pdf]

Dear Reviewers,

thank you for your additional comments and suggestions, we have done our best to amend the manuscript according to them. Please find the detailed answers to your findings below.

Reviewer #1: The author has seamlessly incorporated all the comments. They carefully considered each suggestion and integrated it seamlessly into their revised manuscript.

Thank you again for your valuable notes, we are happy that the revised manuscript meets now your requirements.

Reviewer #2: Authors revised the manuscript according to reviewers comments and suggestions. Manuscript quality is enhanced and its scientific value increased. However, going through the manuscript one can easily spot few lingual and grammatical issues which needs to be addressed. so, authors suggested to get the manuscript proofread by an English native speaker to avoid such issues. Also, authors needs to double check the references style which has to meet with the journal one.

Thank you for this note, an additional language check was performed, the reference style was double checked as well according to the Latex template and the journal standard.

Reviewer #3: Authors revised the manuscript, mostly, according to reviewer comments. My comments for the manuscript:

1. CM-SPAM algorithm is used to first find the frequent sequential pattern in data that are then visualized with proposed three methods (NBVFS-WG, NBVFS-CM and NBVFS-TM). The topic is interesting and the problem is clearly stated. Authors claim that the "proposed visualization methods can significantly lower the time of information processing, for example, during the creation of machine learning models". I did not find discussion about this claim in the results section. Similarly, lines 59-60: "without eliminating any relevant information during post-processing". This line needs some more detail.

Thank you for highlighting these shortcomings. The first statement is discussed more in detail in the Conclusion section, and an additional explanation has been added to the second one (lines 59-60).

2. In abstract: Multiply or multiple?

Thank you for this note, the word has been corrected to multiple.

3. Why CM-SPAM is used as there are other SPM algorithms too? In CM-SPAM, only minsup is the must parameter, other parameter such as min no of patterns, max number of patterns and gap are optional parameters. Authors should discuss why they used optional parameters. These are used to reduce the total number of discovered patterns? It is interesting that max gap of 2 is used. This will enforce the CM-SPAM to find patterns with some gaps and the order of events in the discovered patterns will change. I believe in the two datasets the order is important so why max gap of 2 is used?

Thank you for this comment. The motivation to use CM-SPAM is now discussed on page 8. The max gap was chosen to have an overview on the frequent sequential patterns, based on the results, the value can be changed to get more strict patterns. This depends on the goals of the task and the system. The aim of our work was to demonstrate the applicability area of the method, not to solve a specific problem that would require a more cautious parameter choosing.

By the way add the reference for CM-SPAM.

Fournier-Viger, P., Gomariz, A., Campos, M., Thomas, R. (2014). Fast Vertical Mining of Sequential Patterns Using Co-occurrence Information. Proc. 18th Pacific-Asia Conference on Knowledge Discovery and Data Mining (PAKDD 2014), Part 1, Springer, LNAI, 8443. pp. 40-52.

Thank you for this suggestion, the reference has been added on page 8.

4. Figures quality is very low. Should be replaced with high quality images.

Unfortunately, with tiff format, this is the maximum possible resolution without exceeding the file size limit. Original images are in high resolution pdf and svg format.

5. Add the stats for the two datasets in a table form for easy understanding. Also add their references.

Thank you for this suggestion, a table has been added to page 9, the reference of the click data is presented now on page 8.

6. The data is converted to integer form to apply SPM algorithms. Right? If so then also explain the transformation process. Figures are hard to understand as it is hard to know which integers stands for which event or item.

Right, that is the reason. The transformation is more emphasised now on page 9. The codes and the represented events are discussed on page 8 and 9.

7. This line: For example: the order of 306 actions is always 2 → 3 → 4 → 8; if actions 2,3 and 4 occur, it is almost 100% that 8 307 will occur as well. This kind of rules we can get with sequential rules. How authors are sure that 100% time, 8 will follow 2,3, and 4?

Thank you for this question. You are right, this is an indication to a sequential rule. The method allows to identify these kind of rules without running another algorithm, which is one of its benefits.

8. Ref [2] and [3] seems old. Authors can replace them recent references for the application of SPM. For example:

to extract hypernym relations from texts (Aldine et al. A 3-phase approach based on sequential mining and dependency parsing for enhancing hypernym patterns performance. The Knowledge Engineering Review, 36: E13, 2021.)

for genome analysis (Nawaz et al. Using artificial intelligence techniques for COVID-19 genome analysis. Applied Intelligence, 51: 3086-3103, 2021)

for tourist movement analysis (Cheng et al. A sequential pattern mining approach to tourist movement: The case of a mega event. Journal of Travel Research, 62(6): 1237-1256, 2023)

for workload prediction in cloud environment (M Amiri, L. Mohammad-Khanli and R. Mirandola. A sequential pattern mining model for application workload prediction in cloud environment. Journal of Network and Computer Applications, 105:21-62, 2018)

for mining discrete clinical data (Estiri, S. Vasey and S. N. Murphy. Transitive Sequential Pattern Mining for Discrete Clinical Data. In Proceedings of AIME, pp. 414-424, 2020)

for malware behavior analysis (Nawaz et al. MalSPM: Metamorphic malware behavior analysis and classification using sequential pattern mining. Computers & Security, 118: 102741, 2022)

Thank you for this suggestion, the references have been updated.

9. It would be better if authors provide the GitHub link for the developed code

Thank you for this suggestion, the code will be shared right after the acceptance of the manuscript (as mentioned in the Conclusion section).
