# Supplementary material for: Network-based visualisation of frequent sequences
Source: PLoS One. 2024 May 9;19(5):e0301262. doi: 10.1371/journal.pone.0301262 (PMC11081390; doi:10.1371/journal.pone.0301262)
Supplement: Attachment — Submitted filename: NbVFS_PLos_One_response_letter-round_3.pdf [file pone.0301262.s003.pdf]

Dear Reviewers,

thank you for your additional comments and suggestions, we have done our best to amend the manuscript according to them. Please find the detailed answers to your findings below.

Comment 1. Authors did not provide justification for their claim that how the proposed approach significantly reduce the computational time in information processing

Thank you for pointing on this topic. Our intention was not to claim that the method saves computational time, but it lowers the information processing time of a human. The related parts in the Abstract and in the Conclusion were changed according to this aspect.

Comment 3: Authors should provide the reason for why CM-SPAM is used and not other algorithms
such as CM-SPADE or TKS?

Thank you for this note, the reason of the selection is discussed now on page 8/9. CM-SPADE gives similar performance, TKS does not have the **minsup** parameter.

Comment 7: The pattern 2 3 4 and 8 occurs for which CM-SPAM generates the SUP.
100% occurrences of such patterns means that support is equal to total number of records.
If it is the case then author can keep the line. Otherwise author can rewrite the line to remove the 100% with SUP value.

Thank you for highlighting this potentially confusing part. The mentioned sentence on page 10 has been changed.
